# The Contractility of Isolated Rat Atrial Tissue during Hypoxia is Better Preserved in a High- or Zero-Glucose Environment than in a Normal Glucose Environment

**Published:** 2009-03

**Authors:** Zoltán Szabó, Kristofer Katkits, George Gabro, Rolf GG Andersson

**Affiliations:** 1 *Department of Cardiothoracic, Anesthesia, Linköping Heart Center, University Hospital, Linköping, Sweden;*; 2 *Department of Pharmacology, University Hospital, Linköping, Sweden*

**Keywords:** glucose environment, mechanical function, contractility, hypoxia, glucose, isolated rat atrial myocardium

## Abstract

**Aim:**

Hyperglycemia is known to be associated with an increase in mortality in myocardial infarction and intensive care patients despite the fact that glucose metabolism plays a central role in myocardial protection. We studied the effect of different glucose levels (22 mM L^-1^; 5.5 mM L^-1^; and 0 mM L^-1^) on the contractile reserve of isolated rat atrial myocardium during and after hypoxia.

**Methods:**

We observed the contraction of isolated rat atrium strips caused by electrical-field stimulation in a modified Krebs-Henseleit Buffer (KHB) organ bath oxygenated with 95% O_2_ + 5% CO_2_ at 37°C. We applied two periods of hypoxia and two periods of reoxygenation. Three glucose concentrations were used in the buffer to study the effect of glucose (high- n=6; normal- n=7; and zero-glucose n=6). The effect of isoproterenol 1 μM L^-1^ was tested during the second ischemic period.

**Results:**

The main finding was that both a zero-glucose (27.8 ± 5.9 vs. 14.7 ± 3 % of baseline tension) and a high-glucose environment (38.5 ± 14 vs. 14.7 ± 3 % of baseline tension) had a positive effect in terms of better contractility than the normal-glucose buffer during both the first (p=0.00062) and the second ischemic period (31.2 ± 5.9 % zero-glucose vs. 14.7 ± 4.2 normal-glucose vs. 35.3 ± 15.9% high-glucose p=0.0038).

**Conclusion:**

Both zero-glucose and high-glucose environments resulted in a better contractile reserve in isolated rat atrial myocardium during hypoxia than in a normal one. The exact clinical relevance of this observation is, at present, unclear.

## INTRODUCTION

The question: is hyperglycemia advantageous or disadvantageous in critically ill patients is currently being reassessed and intensely debated (1-3). Clinical data has emerged suggesting that hyperglycemia increases mortality in intensive care patients and patients with ischemic heart disease, especially those with diabetes and myocardial infarction (4, 5).

However, hyperglycemia may be only an indicator of extreme stress as proposed by Hiesmayr (6). Extreme changes in blood glucose concentration are a central feature in the clinical practice of cardiac anesthesia; on the one hand hyperglycemia and on the other hypoglycemia caused by intraoperative insulin treatment, which may be devastating in anesthetized patients where the clinical signs of hypoglycemia are concealed. Clinical data showing increased mortality associated with high blood glucose levels are apparently contradicted by in-vitro studies demonstrating that high glucose levels have a beneficial effect on the ischemic myocardial tissue itself. *In vitro* experiments show better survival for myocardial cells exposed to hypoxia, even in diabetic myocardial cells (7), due to preserved anaerobic ATP production from glucose. These facts are in concordance with Hiesmayr´s observation.

Atrial myocardial protection is a new issue (8). It is common knowledge that atrial fibrillation may diminish cardiac output up to approximately 30%. Atrial fibrillation is a common clinical feature (in 29.5% of patients) after cardiac surgery and may double mortality (9).

The association of high glucose levels with mortality in intensive care unit (ICU) patients has never been proved, and postoperative mortality in coronary patients after cardiac surgery has mainly cardiac causes (10). It seems that the correlation between glucose concentration and the clinical picture is a complex issue with many mechanisms involved.

In this study we looked to see if post-ischemic atrial mechanical function is influenced by the glucose concentration before and during ischemia. The primary aim of this study was to clarify the effect of zero-, high- and normal-glucose conditions on the post-ischemic mechanical function of rat atrial strips. The change in capacity to generate tension during hypoxia was used to characterize myocardial contractile reserve (11).

## METHODS

The local ethics committee approved the study. Hearts from healthy male Sprague-Dawley rats (weight 250g) with free access to food and water and light/dark on a 12-hour basis, were used in the study.

### Preparation of myocardial strips

The rat heart was removed immediately after sacrifice. The atrial muscle was divided along its fiber lengths into strips measuring 0.2 × 3 × 10 mm (1-3 strip per atrium). A smaller cotton string loop was tied to one end of the strip, and an approximately 10-15 cm length of string to the other end.

### The organ baths and measurements

The strips were then attached to a fixed point at the loop end and to the tension-transducer at the string end and submerged in oxygenated (1.5 L/min 95% O_2_ and 5% CO_2_) modified KHB (Na^+^ 137 mM, K^+^ 6 mM, Mg^2+^ 1.3 mM, Ca^2+^ 2.2 mM, Cl^-^ 134 mM, HCO^3-^ 15,4 mM, H_2_PO^4-^ 1.2 mM) containing glucose 5.5 mM (normal-glucose environment), 22.0 mM (high-glucose environment) or 0 mM glucose (zero-glucose environment) at 37°C in three organ baths simultaneously.

The strips were stimulated by a Grass S88 stimulator using silver electrodes (1 msec, 2 Hz, 40 - 60 mV) to induce regular maximal contractions. The contractions were recorded continuously by an isometric strain gauge transducer (Grass Model FT. 03, Quincy, MA, USA) coupled to a Grass polygraph. The device was calibrated before each series of measurements.

### Experimental protocol

**Figure 1:**
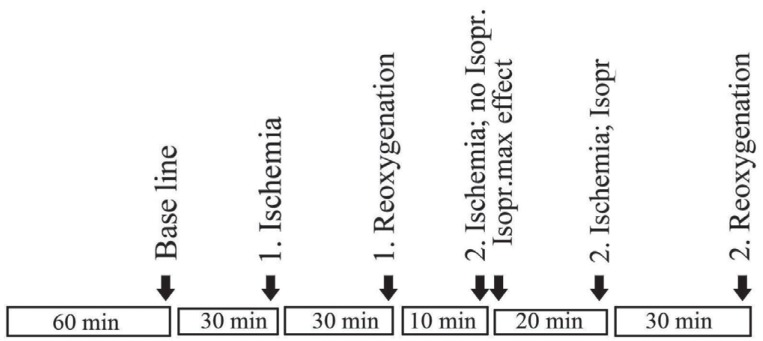
The experimental protocol.

The experiments always began after a stabilization period of 60 minutes as shown in the time protocol presented in Figure 1.

*Baseline contractions (prehypoxia)* for each preparation were measured after which the gas mixture was shut off (*first hypoxia*) for 30 minutes.

New measurements were made followed a period of *first reoxygenation* for 30 minutes during which the gas mixture was turned on again.

New measurements representing first reoxygenation were made at the end of this period.

Thereafter the gas was once again shut off, and 10 minutes into the *second hypoxia period*, the β-agonist isoproterenol was added to a final bath concentration of 1 μML^-1^. Two minutes later measurements, noted as isoproterenol maximum effect, were made.

After another 20 minutes of hypoxia with isoproterenol new tension measurements were made (*30 minutes of hypoxia and 20 minutes with isoproterenol*) after which the baths were once again reoxygenated for the last time.

After 30 minutes into *the second reoxygenation period*, the tension measurements were noted and the experiment terminated. Details of the protocol are shown in Figure 1.

*Numbers of samples were:* high- *n = 6*, normal- n = *7* and zero-glucose n = 6.

The effects of hypoxia and reoxygenation were measured as contractility under the described conditions and presented as % contraction compared to baseline level (prehypoxia) as shown in the results section. The amplitude of contraction was interpreted as myocardial (loss of) function, thus being an indicator of myocardial tissue damage.

### Statistical methods

The data are presented as mean values ± SD. The comparisons between groups were done with, ANOVA multiple measurements design combined with Tukey honest significant difference test for post hoc testing. The p value <0.05 was considered significant.

We used a Hewlett Packard personal computer with an operative system Windows XP 2005 and Statistica 8. (StatSoft®, Tulsa, OK, USA) to calculate the results.

## RESULTS

**Figure 2:**
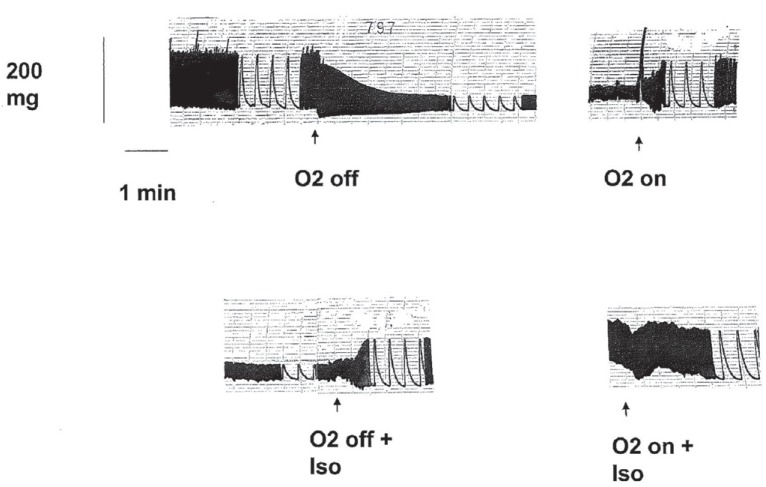
A typical registration from a preparation in normal-glucose environment (5.5 mM), during the second hypoxia period, the β-agonist isoproterenol (Iso) was added to a final bath concentration of 1 μML^-1^.

A typical registration from a preparation in normal-glucose environment is shown in Figure 2.

The baseline tension values we compared measurements to were: in a high-glucose environment 228 ± 244 mg (n=6); normal-glucose environment 241 ± 114 mg (n=7); zero-glucose environment 288 ± 188 mg (n=6) the difference between them according to ANOVA had a p value of 0.841.

Using the Tukey honest significant difference test, results showed a significantly better contraction in both high-glucose 38.5 ± 14% of baseline tension (p=0.0005) and zero-glucose conditions 27.8 ± 5.9% of baseline tension (p=0.039) compared to normal-glucose conditions (14.7 ± 3% of baseline tension) during the first ischemic period. No difference between the high and zero glucose groups was found.

During the second ischemic period the corresponding figures were 31.2 ± 5.9% for zero-glucose vs. 14.7 ± 4.2 (p=0.022) for normal-glucose vs. 35.3 ± 15.9% for high glucose (p=0.0048). No difference between the high and zero glucose groups was found at this time.

For within group differences see Table 1.

**Table 1:** Contractility in three different glucose concentrations during hypoxia and reoxygenation in % related to basal measurement mean ± SD

Group/sampling	Glucose = 0	Glucose=5.5 mM	Glucose=22.0 mM	Multiple
	N=6	N=7	N=6	meas.
	Contractility%	Contractility%	Contractility%	ANOVA

O2off (1.hypoxia 30 min)	*27.8 ± 5.9*^a^	14.7 ± 3	*38.5 ± 14*^c^	p=0.00062
O2on (1.reoxygenation 30 min)	*62 ± 3.3*†	*55 ± 14.8*†	*70.8 ± 18.7*††	p=0.158
O2off (2.hypoxia10min)	*31.2 ± 5.9*^a^	*14.7 ± 4.2*	35.3 ± 15.9^b^	p=0.0038
O2off + Iso max	78 ± 15.8	*71.4 ± 28.2*†	*65.3 ± 24.2*	p=0.679
O2off + Iso (20 min)	*29.5 ± 6.8*†	*21.3 ± 7.2*	39 ± 19.6	p=0.064
O2on + Iso (30 min)	72 ± 18.3	64.4 ± 12.2	*85.1 ± 32.4*††	p=0.266

The baseline tension values we were comparing all measurements to were: in the high-glucose environment 228 ± 244 mg (n=6); normal-glucose environment 241 ± 114 mg (n=7); zero-glucose environment 288 ± 188 mg (n=6). Inter-group differences:

a p<0.05;

b p<0.01;

c p<0.001.

Within group differences related;

† <0.05;

†† p<0.01;

Cursive and cursive underlined is to mark the within group differences at different measuring times.

## DISCUSSION

The main finding in this study was that during hypoxia both zero-glucose and high-glucose environments were more protective than normal glucose expressed as tension generation in isolated rat atrial myocardium strips during hypoxia. This is under the presumption that the change in the capacity to generate tension under hypoxia reflects myocardial contractile reserve (11).

It is possible that a high glucose concentration stimulates anaerobic glycolysis creating a glycogen store that under ischemic conditions maintains the ability to restore the contractility (6). The role of potassium sensitive ATP channels and glycolysis as described previously by Jovanovich in isolated cardiomyocytes (12) may also be involved in the ischemic protection demonstrated in our case. There are data showing that glucose protects embryonic cardiac cells against hypoxia in terms of increased glucose uptake and decreased cell death (13).

Fasting protects ischemic heart from injury in isolated rat heart, probably due to glycogen utilization caused by an increase in the active form of glycogen phosphorylase and an increase in the cytosolic redox state (14). Hypoglycemia was easiest to achieve by long starvation (15). However our animals were not fasted. A further explanation may be that AMPK (AMP activated protein kinase) is generally quiescent under normal conditions but is activated in response to hormonal signals and stresses sufficient to produce an increase in AMP/ATP ratio, such as hypoglycemia, strenuous exercise, anoxia and ischemia. Once active, muscle AMPK enhances uptake and oxidative metabolism of fatty acids as well as an increase in glucose transport and glycolysis (16). Preconditioning also could have contributed to the improvement after first ischemia (17). Endogenous amino acids may also have played a protective role as occurs in insulin coma (18). In this study we have shown that neither high- nor zero-glucose conditions are detrimental for isolated rat atrial myocardium strip contractility during hypoxia. The exact metabolic protection mechanism during hypoxia in a zero-glucose environment is an open question. There are few publications on the effect of zero-glucose on isolated myocardium during hypoxia and none of these can explain our findings (19, 20).

As atrial function may be life-saving in the postoperative period following cardiac surgery, active atrial myocardial protection during cardioplegic arrest, as in open heart surgery, may possibly improve postoperative survival. Based on our study we cannot discriminate between the roles of different hypotheses. These results from a clinical point of view are only an observation. The results of this study are experimental observations and shed no light on the mechanisms that lie behind.
